# Immunotherapy of Ovarian Cancer: The Role of Checkpoint Inhibitors

**DOI:** 10.1155/2015/191832

**Published:** 2015-07-07

**Authors:** Francesca De Felice, Claudia Marchetti, Innocenza Palaia, Daniela Musio, Ludovico Muzii, Vincenzo Tombolini, Pierluigi Benedetti Panici

**Affiliations:** ^1^Department of Radiotherapy, Policlinico Umberto I, “Sapienza” University of Rome, Viale Regina Elena 326, 00161 Rome, Italy; ^2^Department of Gynecological and Obstetrical Sciences and Urological Sciences, “Sapienza” University of Rome, Viale del Policlinico 155, 00161 Rome, Italy

## Abstract

Ovarian cancer is the most important cause of gynecological cancer-related mortality, with the majority of women presenting with advanced disease. Although surgery and chemotherapy can improve survival rates, it is necessary to integrate alternative strategies to improve the outcomes. Advances in understanding the role of immune system in the pathogenesis of cancer have led to the rapid evolvement of immunotherapy, which might establish a sustained immune system response against recurring cancer cells. Recently, it has emerged that powerful immunologic effector cells may be blocked by inhibitory regulatory pathways controlled by specific molecules often called “immune checkpoints,” which turn off the immune system. Similarly, cancer cells are able to use these checkpoints to avoid immune control and rejection. Inhibition of these inhibitory pathways represents a potent strategy in the fight against cancer and is currently under investigation with encouraging results in some cancers, such as melanoma. In ovarian cancer researches are still in an early phase, but with promising results. In this review we will explore the rationale of immunotherapy in ovarian cancer with a special focus on these emerging molecules.

## 1. Introduction

*Overview*. Ovarian cancer (OC) is a common condition in women scenario and it represents the principal cause of death from gynaecologic cancer in United States [1]. It is estimated that 21.290 cases will have been diagnosed in 2015 and that 14.180 women will have died due to this malignancy. About 90% of OC are epithelial carcinomas and 70% of those have a serous histology [2]. Death rate from OC declined from 1970s to 1990s but it has since then remained stable.

In light of these discouraging data, the development of novel therapies for OC has become a priority. Recent better molecular characterization and immune system identification are the starting point of future research in immunotherapy.

Undoubtedly, the next decade will see immunotherapy coming to the clinic use alongside standard regimes and it is possible that it could replace cytotoxic chemotherapy in combination strategies. Therefore, in addition to possessing expertise with immunotherapy, oncologists will be expected to conduct trials of novel agents in combination with standard treatment. Thus, it is paramount to focus the attention on maximising the knowledge of the more important component of the immune system. Loss in this challenge could run the risk that oncologists will take a passive role in the development of new strategies.

This paper reviews the rationale for immunotherapy and the main approaches under investigation in OC, with a special focus on the role of checkpoint inhibitors.

We will briefly describe the human immune system in an attempt to provide a means of understanding how it relates specifically to the clinical practice.

## 2. Immune System and Cancer Disease

### 2.1. The Father of Immunotherapy

The role of immunotherapy in cancer treatment has been identified decades ago, because of beneficial effect of severe induced infection on tumour regression. At that time, Coley showed that inoculation with streptococcal organisms resulted in the shrinkage of inoperable bone and soft-tissue sarcomas [3]. However, severe criticisms due to the inconsistency of the method and results emerged in the scientific community, across the years. One explanation was that other physicians, who tested his treatment, did not report the same excellent effect. These results, as well as the concurrent development of radiotherapy and chemotherapy, determined immunotherapy to slowly disappear from treatment cancer scenario [4]. Since Coley's death, immunology has represented an active research field and, nowadays, immunotherapy is considered again a valid treatment option in different types of cancer [5].

### 2.2. Basic Knowledge of Human Immune System

The human immune system can be divided in two components: the innate and the adaptive immunity.

The innate immune system consists of natural killer (NK) cells, dendritic cells, and macrophages and neutrophils, whereas B cells and T cells, including cytotoxic (CD8+ T or CTL) cells, helper (CD4+ T) cells, and NK T cells, are specific of the adaptive immunity [6]. The innate immunity provides a first line response against pathogens in a nonspecific manner; it has no immunologic memory and it is not able to recognize antigen. Thus, in terms of tumor immunology, its contribution is marginal and limited to secreted cytokines that recruit immune cells. On the other hand, the adaptive immune system plays a central role in the antitumor immune response, due to its capability in processing “nonself” cells. T cell activation requires at least two conditions. Firstly, the presentation of an antigen to a T cell receptor (TCR) by a major histocompatibility complex (MHC) molecule on antigen presenting cells (APCs). Secondly, the interaction of the CD28 receptor on T cells to B7 costimulatory molecules (B7-1 and B7-2) on APCs [7].

Actually, the immune system often recognized the tumor cells as “self,” because they are basically expression of patient's own cell types. The distinction between “self” and “nonself” is provided by cancer-specific antigens that are expressed by tumor cells. Tumor antigens are traditionally divided in two classes: tumor specific antigens (TSAs) and tumor associated antigens (TAAs). TSAs are exclusively expressed by tumor cells and thus they are easily recognized as “nonself” by the immune system. TSAs represent an ideal target for anticancer immunotherapy. On the contrary, TAAs are normally found on nonmalignant cells. For instance, oncospermatogonal antigens are expressed by tumor cells as well as normal spermatocytes; carcinoembryonic antigen (CEA) is expressed on fetal tissues and in several cancer types. Therefore TAAs are less expected to activate an effective and efficient immune system response [8].

### 2.3. Immunoediting Process: The 3Es of the Immune System

Theoretically, the immune system recognizes nascent transformed cells, in order to prevent progression to clinical tumor. If intrinsic tumor suppressor mechanisms failed, the cancer immunoediting is engaged. It is an extrinsic tumor suppressor mechanism that consists of 3 sequential phases: elimination, equilibrium, and escape [9].

The elimination phase was previously known as cancer immunosurveillance. In this phase, transformed cells are recognized and eradicated by the innate and adaptive immune system. CD8+ T cells, CD4+ T cells, natural killer (NK) cells, and NK T cells secrete interferon- (INF-) *γ* to inhibit tumor cell proliferation and angiogenesis, whereas macrophages and dendritic cells are processed to phagocytise and remove tumor cells killed [10].

The cells that are not eliminated in this phase may then enter the equilibrium phase, in which their development is prevented by adaptive immunologic mechanism. CD8+ T cells and dendritic cells secrete INF-*γ* and interleukin- (IL-) 12, respectively, and preserve tumor cells in a steady state. This is a functional state in which latent tumor cells are specifically controlled by the adaptive immunity. This dynamic balance can persist for long period, sometimes exceeding 20 years [9].

In response to immune system, tumor cells can change their characteristics in immune resistant cells and therefore escape from immune system suppression. In this final phase, tumor cells emerge and become clinically apparent, because they are no longer blocked by immunity. The generation of immune resistant tumor cells can occur in several ways: through loss of tumor antigens expression; through downregulation of MHC; through the overactivation of the prooncogenic transcription factor STAT3; through the overexpression of antiapoptotic effector BCL-2; through the expression of inhibitory cell surface molecules, such as programmed cell death 1 ligand 1 (PD-L1), cytotoxic T-lymphocyte associated protein-4 (CTLA-4), and Fas ligand (FasL), which directly kill cytotoxic CD8+ T cells. Otherwise tumor cell escape can be a consequence of an immunosuppressive state established in the tumor microenvironment. This condition may result from the secretion of immunosuppressive cytokines, like IL-4, IL-1*β*, vascular endothelial growth factor (VEGF), and prostaglandin-E2 (PGE2), which recruit regulatory cells. Particularly, the secretion of IL-4 recruits macrophages that inhibit CD4+ T cells, by expressing transforming growth factor-*β* (TGF-*β*), IL-10, and platelet-derived growth factor (PDGF), whereas the secretion of IL-1*β*, VEGF, and PGE2 determines the accumulation of myeloid-derived suppressor cells that blocks T cell function [11].

### 2.4. The Rationale

Over the last decade immunotherapy has become a mainstay in anticancer therapy. The aim is to eradicate tumor cells stimulating the normal human immune system. We need to integrate the potential understanding of the immunoediting process from the 3Es and the tumor characteristics to conduct the optimal treatment. It is difficult to define a clear role of immunotherapy; nonetheless it is reasonable to hypothesise that any immune molecule capable of activating this process might have a useful role in eradication of nascent tumor cells. At this time it is paramount that oncologists are familiar with the immunoediting process so that they can have a role in the rational development of innovative clinical trials. Immunotherapy has the potential to guide the future direction of cancer treatment.

The stabilization of equilibrium state, as well as the inhibition of tumor escape mechanisms, should be clinical endpoints.

## 3. Cancer Immunotherapy: The Role of Immune Checkpoint

### 3.1. Current Immunotherapy Options

Current immunotherapies for cancer treatment include therapeutic vaccines, cytokines, immune modulators, immune checkpoint inhibitors, and adoptive T cell transfer [12].

Therapeutic vaccines are designed to treat established cancers and may be used in the induction of the tumor-directed immune response of the patients through the introduction of tumor antigens. The other approaches such as immune checkpoint inhibitors and adoptive T cell transfer are designed to augment anticancer immunity against cancer [13].

### 3.2. Focus on Immune Checkpoint

Nowadays, one of the most promising strategies seems to be the takeover of immune cell-intrinsic checkpoints that are induced on T cells activation. The blockade of one of these checkpoints, such as CTLA-4 [14] or the programmed death 1 (PD-1) receptor, has recently been found to be active to achieve an immune-modulation approach in the treatment of solid tumors [15, 16]. The immune checkpoint blockade targeted agents might represent breakthrough drugs in the treatment of solid tumors and have generated greater expectations in the field of cancer immunotherapy, even in OC [17, 18].

### 3.3. Mechanisms of Action of Immunomodulators

T cells activity is regulated by a great number of different molecules, as well as immune-modulatory signals, both costimulatory and coinhibitory [19]. To avoid inappropriate T cell activation, resulting in autoimmunity, negative regulators of T cell immunity, including CTLA-4 and PD-1, are needed. Preclinical models on the blockade of these coinhibitory molecules showed an antitumor immune response [17]. In fact both CTLA-4 and PD-1 are key immune checkpoint proteins and represent a further promising immunotherapeutic target.

CTLA-4 is a member of the CD28:B7 immunoglobulin superfamily, typically low-expressed on the surface of naive effector T cells and regulatory T cells (Tregs) [20]. When naive T cells are stimulated through the TCR, CTLA-4 is upregulated and competes with CD28 for B7 and, finally, determines the suppression of T cell activity [21]. It was found that the antitumor effect of CTLA-4 blockade might be obtained also by depletion of Treg [22], as revealed in a model of mouse melanoma, in which both the augmentation of T effector cell function and inhibition of Treg activity through the blockade of CTLA-4 manage to obtain a strong antitumor response.

PD-1 is expressed on chronically stimulated T cells, as well as Tregs, activated B cells, and NK cells [23]. Differently from CTLA-4, which regulates T-lymphocytes at the level of initial activation, PD-1 regulates immunity at multiple phases of the immune response, including its effect on effector T-lymphocyte activity in the peripheral tissues. Experimental models showed that PD-1 deficient mice present enhanced immunity with phenotypes characterized by autoimmune cardiomyopathy and a lupus-like syndrome [24, 25].

The activity of PD-1 is related to its interaction with its ligands, PD-L1 (B7-H1) and PD-L2 (B7-DC) [26]. Both ligands, especially PD-1, are expressed on many hematologic and nonhematologic human tumors [27].

Generally, in human cancer, when PD-1 binds with cells bearing one of its ligands, T cell activity is attenuated (phenomena known as peripheral tolerance), which prevents these T cells from rejecting the tumor at the tissue level, and tumors can thereby employ the PD-1 inhibitory pathway to silence the immune system [28].

## 4. Clinical Trials with Immune Checkpoint Blockade Targeted Agents in OC

Based upon the findings of preclinical studies, suggesting the involvement of these molecules in immune control, various agents blocking CTLA-4, PD-1, or PD-L1 or other immune molecules are currently investigated in ovarian cancer (OC) treatment. Details are shown in Table 1.

### 4.1. Anticytotoxic T-Lymphocyte Antigens

The CTLA-4 is currently being investigated as a single or combinatorial therapy in clinical trials involving several cancer types.

Ipilimumab and tremelimumab are fully human IgG1 or IgG2 antibodies, respectively, that antagonize the CTLA-4 immune checkpoint. The majority of clinical data derived from studies in patients with melanoma. In these studies CTLA-4 blockade has yielded objective responses to such an extent that ipilimumab was FDA approved to treat metastatic or unresectable melanoma in 2011 [29, 30]. It represented the first standard-of-care immune checkpoint inhibitor.

Experience in OC is actually based on small population studies but results seem to be interesting.

Hodi et al. firstly showed [31, 32] antitumor effects in patients with stage IV OC patients. Initially [31], they reported that a single infusion of ipilimumab (3 mg/kg) in two-stage IV OC patients previously vaccinated with granulocyte-macrophage colony-stimulating factor modified irradiated autologous tumor cells (GVAX), was well tolerated, and triggered a decrease or stabilization of CA-125 levels of several months' duration.

In order to clarify the toxicity and antitumor efficacy, they treated additional 9-stage IV OC subjects by using the same antibody dose and schedule (with the exception of one patient) [32]. In one patient, an objective radiographic response was noted and multiple infusions of anti-CTLA-4 antibody every 3 to 5 months have maintained disease control over 4 years; furthermore, 3 out of 9 patients had stable disease of 6 (ongoing at the moment of paper's publication), 4, and 2 months' duration, as measured by CA-125 levels and radiographic criteria, in the absence of serious toxicities.

Few patients showed manageable inflammatory toxicities. Tumor regression correlated with the CD8+/Treg ratio, suggesting that other forms of therapy that target Treg depletion might have a synergistic effect when combined with the tumor vaccine and CTLA-4 antibody molecules.

These findings prompted a phase II clinical trial to evaluate ipilimumab as monotherapy in platinum-resistant OC patients (NCT01611558) [33]. Ipilimumab can cause significant immune-related adverse events (AEs), and the more common observed side effects include diarrhea, colitis, and dermatitis. Less common severe immune-related adverse events include hypophysitis, thyroiditis, and hepatitis.

Tremelimumab (previously known as ticilimumab) is a fully human IgG2 monoclonal antibody to CTLA-4. In contrast to ipilimumab, a large phase III trial [34] in melanoma did not demonstrate improved PFS or OS compared with cytotoxic chemotherapy although durable responses were observed in some patients. Much speculation have been done about the potential reasons for this clinical result, because both phase III clinical trials [14, 29] testing ipilimumab succeeded in showing improved OS. It has been proposed that human IgG1 (the ipilimumab subclass) binds with a higher affinity to Fc*γ*Rs than human IgG2 (the subclass of tremelimumab) does [35], therefore suggesting that tremelimumab might determine a CTLA-4 antibody mediated Treg-cell depletion to a lesser extent [36, 37]. The combination of tremelimumab and a PD-1 inhibitor (see below) is currently ongoing, in a phase I study including ovarian and cervical cancer patients [38].

### 4.2. PD-1 and PD-L1 Targeting Agents

The therapeutic benefit obtained with CTLA-4 inhibition led to the effort in identifying other potential immune checkpoint inhibitors that should have been more specific and equally efficacious and have less immune toxicity. PD-1 and PD-L1 inhibitors were identified as potentially accomplishing those requirements. Differently from CTLA-4, which regulates T-lymphocytes at the level of initial activation, PD-1 regulates immunity at multiple steps, including exerting its effect on effector T-lymphocyte activity in the peripheral tissues. Several monoclonal antibodies have been developed that block the PD-1 system, either by interactions with the PD-1 receptor or with its specific ligands.

Nivolumab (also known as BMS-936558 or MDX1106) is a fully human IgG4 monoclonal antibody that targets PD-1. A phase I/II clinical trial [39] tested the safety and efficacy of nivolumab at doses of 0.1 to 10.0 mg/kg of body weight intravenously every 2 weeks for up to 12 cycles until disease progression or a complete response occurred. Patients with advanced melanoma, non-small-cell lung cancer, prostate cancer, renal cancer, and colorectal cancer were enrolled. Among the 296 patients, those with metastatic melanoma achieved the higher rates of objective responses (27.6%), with a median OS of 16.8 months; conversely responses were not observed in colon and prostate cancer patients. Responses were seen in both PD-L1 positive and negative patients, even if with lower extent. Common treatment-related adverse events included fatigue, diarrhea, pruritus, rash, nausea, and decreased appetite. Grade 3 or 4 treatment-related adverse events were seen in 14% of patients. Treatment-related serious adverse events were noted in 11% of patients and included pneumonitis (3%, and grade 3 or 4 in 1%), colitis, hepatitis, thyroiditis, and hypophysitis.

Recently, at the 2014 ASCO meeting [40], the first clinical trial of nivolumab treatment against platinum-resistant OC has been presented. A total of 18 evaluable patients were treated with nivolumab: 10 patients were administered 1 mg/kg and 8 patients were administered 3 mg/kg, each every 2 weeks for 1 year. Starting at week 8, patients were assessed every 8 weeks and patients with disease progression were taken off study. Median treatment duration was 14 weeks. There were two serious treatment-related AEs: one patient in the 1 mg/kg group experienced grade 3 fever, disorientation, and gait disorder and one patient in the 3 mg/kg category experienced grade 3 fever and deep-vein thrombosis. Other grade 3/4 treatment-related AEs included hypothyroidism (two patients, both in the 1 mg/kg group); heart arrhythmia (one patient, in the 3 mg/kg group); and lymphocytopenia (one patient, in the 1 mg/kg group). Interestingly, the overall objective response rate was 17%. The 3 mg/kg dose may be more favourable (25%) than 1 mg/kg (10%). Two patients in the 3 mg/kg group experienced complete response (CR; response rate 25%). Among those receiving 1 mg/kg nivolumab, one experienced a partial response (10% response rate) and two patients experienced stable disease (SD). Further researches are investigating biomarkers predicting response.

A further molecule investigated in OC is pembrolizumab (MK-3475, formerly known as lambrolizumab), a humanized IgG4 monoclonal antibody against PD-1. It was found to be active in treating both melanoma and NSCLC [41–43], similar to nivolumab. Actually, no randomized trial has compared the two agents, which are surely different in binding affinities, nivolumab being a fully human IgG4, and pembrolizumab is humanized. Currently, phase I trials are ongoing with both molecules including OC patients. Recently, an interim analysis with pembrolizumab showed preliminary signal for clinical efficacy in recurrent OC [44].

In addition to antibodies targeting PD-1, several different anti-PD1-L1 monoclonal antibodies, such as BMS-936559, MPDL3280A, MEDI4736, and MSB0010718C, have been developed which might enhance immune function. It was found that the ligand/receptor interaction inhibits the T-lymphocyte response by inhibiting the kinases involved in T-lymphocyte activation via phosphatase activity and other signaling pathways [45].

BMS-936559 is a high-affinity, fully human IgG4 monoclonal antibody that binds PD-L1 and that blocks PD-L1 from binding its two known receptors PD-1 and CD8.

It was safe in a phase I trial that included 17 OC patients [15] in escalating doses of 0.3–10 mg/kg iv every 14 days in 6-week cycles for up to 16 cycles or until the patient had a complete response or confirmed disease progression and observed durable tumor regression and prolonged stabilization of disease. Common side effects included fatigue, infusion reactions, diarrhea, arthralgia, pruritis, rash, nausea, and headache. In the trial, only OC patients at the 10 mg/kg dose achieved objective responses: 1 (6%) with a partial response and 3 (18%) with stable disease lasting more than 24 weeks.

MSB0010718C is a fully human IgG1 monoclonal antibody targeting PD-L1. Unlike other PD-L1 targeting agents, it is a native Fc receptor, allowing for antibody dependent cell mediated cytotoxicity.

In a phase I trial [46] 27 patients with refractory malignancies were treated with MSB0010718C at 1, 3, 10, and 20 mg/kg twice weekly. Eleven patients in the study had received prior treatment with an immunotherapy. At the 3 and 10 mg/kg doses, the drug was found to inhibit 93.8% and 93.2% of the PD-L1 receptor on peripheral leukocytes. Additionally, a linear PK profile was found, with a maximum concentration of the drug achieved at 1.5–2 hours following infusion. At the 20 mg/kg dose, a dose-limiting immune-related adverse event was noted. In this trial also OC patients were included and, interestingly, a larger subsequent meta-analysis of the company developing the drug (Merck) [47], including 23 patients' OC cohort, showed 48% of patients reaching stable disease and 17% getting a partial response within 30 weeks of treatment start, though 13 had been taken off the drug. Noteworthily, the responses came despite 77% of patients having already failed at least three lines of therapy. More recently [48], efficacy data from the 23 patients followed up for more than 2 months (range 2–8 months) were presented. Four patients (17.4%) achieved an unconfirmed partial response, 11 (47.8%) patients had stable disease, and 2 patients had >30% tumor shrinkage after progression. Median PFS was 11.9 weeks and the PFS rate at 24 weeks was 33.3%. Toxicity was manageable and only 2 patients (8.7%) experienced grade ≥ 3 drug-related AEs. The most commonly reported AEs were fatigue, nausea, and diarrhea. A larger, phase II clinical trial enrolling 590 patients is ongoing.

Other molecules, such as MPDL3280A and MEDI4736, are currently investigated in phase I trials including OC patients.

### 4.3. Concluding Remarks

OC is defined as an immunogenic tumor that exhibits a spontaneous antitumor immune response [49]. Tumor tissue can be considered a Darwinian microenvironment that selects the better strategy to elude the immune system. Immune checkpoint pathways are modulated by ligand/receptor interactions. Expression of specific ligands, such as PD-L1 and CTLA-4, in the stroma or in the tumor cells associated, is paramount to improve growth and resistance to immune attack. It depends on both tumor type and histology, and therefore it also represents the major limitation of immunotherapy. Maybe the identification and characterization of similar patients population, as well as tumor histology, could provide data to facilitate the development of novel treatment strategies. Immune check point inhibitors may have a synergic mechanism in multimodality treatment and thus a positive effect on overall survival, with a tolerable toxicity profile. Further randomized trials are paramount to prospectively clarify this hypothesis.

## 5. Conclusions

In the last years, immunotherapy has achieved an important role in the fight against cancer and also, in OC immunological phenomena, has been demonstrated to play a central role.

Novel and promising agents have been developed. Immune checkpoint inhibitors have shown clinical activity in several cancers, especially melanoma, and they represent a major step forward in the fight against cancer.

These novel therapies will likely play a role also in OC given the potential for rapid, durable responses and their favourable toxicity profiles. Their function in the treatment of patients with OC remains to be defined but initial results seem to be promising. Next challenges should be the clinical development of combinatorial approaches and further defining patients who benefit from immune checkpoint monotherapy and patients who require potentially more active albeit more toxic combination regimens. Finally, the definition of potential biomarkers that can determine which immune checkpoint pathway or pathways dominate in a particular tumor will be crucial to guide the choice of inhibitor.

The possibility of using immunotherapy in OC is still restricted to clinical trials but it is reasonable to expect that over the next years important advances in OC immunotherapy will be made, running further phase II and III trials development.

## Figures and Tables

**Table 1:** Active trials of checkpoint inhibitor in ovarian cancer.

Drug	Antibody type^*∗*^	Notable side effects	Study (phase)
Anti-CTLA-4 antibodies			
Ipilimumab	IgG1	Diarrhea, colitis, fatigue, transaminitis, hypophysitis.	I; II
Tremelimumab	IgG2	Diarrhea, fatigue, nausea, vomiting, anorexia, rash	I

Anti-PD-1 antibodies			
Nivolumab	IgG4	Pneumonitis, lymphopenia, fatigue, diarrhea, hepatitis, renal insufficiency	I; II
Pembrolizumab	IgG4-kappa	Pneumonitis, fatigue, thyroid problems	I

Anti-PD-L1 antibodies			
BMS-936559	IgG4	Fatigue, hyperglycemia, infusion reaction, endocrinopathies, adrenal insufficiency, myasthenia gravis	I; II
MEDI4736	IgG1-kappa	Diarrhea, fatigue, rash, vomiting	I
MPDL33280A	IgG4	Hyperglycemia, hypophysitis, pericardial effusion, fatigue	I
MSB0010718C	IgG1	Laboratory abnormalities, creatine kinase increase, myositis, myocarditis	I; II

^*∗*^All fully human, except pembrolizumab which is a humanized IgG4-kappa.

Updated on February 28, 2015.
